# Pembrolizumab-Associated Immune-Related Cystitis Mimicking Urothelial Malignancy as Part of a Multi-organ Immune-Related Adverse Event (irAE) Phenotype in a Patient With Metastatic Squamous Cell Lung Cancer: A Case Report and Literature Review

**DOI:** 10.7759/cureus.115708

**Published:** 2026-09-03

**Authors:** George Habib, Elaria M Habib, John Diks

**Affiliations:** 1 Pharmacy, Cabell Huntington Hospital, Charleston, USA; 2 Pathology, Marshall University Joan C. Edwards School of Medicine, Huntington, USA

**Keywords:** case report, hematuria, high-grade urothelial carcinoma, immune checkpoint inhibitor, immune-related adverse events, immune-related cystitis, non-bacterial cystitis, non-small cell lung cancer, pembrolizumab, urine cytology

## Abstract

Immune checkpoint inhibitors (ICIs) have revolutionized the treatment of metastatic non-small-cell lung cancer (NSCLC) but can cause a broad spectrum of immune-related adverse events (irAEs) affecting multiple organ systems. Although common irAEs are well characterized, immune-related cystitis is rare and may pose a significant diagnostic challenge, particularly when urine cytology demonstrates atypical urothelial cells that mimic high-grade urothelial carcinoma. We present the case of a 55-year-old man with metastatic squamous cell carcinoma of the lung who developed hematuria and non-bacterial cystitis during pembrolizumab therapy in the setting of multiple other irAEs, including hypophysitis resulting in secondary adrenal insufficiency and hypothyroidism. Following the onset of hematuria, computed tomography (CT) of the abdomen and pelvis demonstrated an acute cystitis picture. Importantly, there was no evidence of urinary calculi or progression of pelvic metastatic disease to otherwise explain his symptoms. Urine cytology subsequently showed marked urothelial atypia concerning for high-grade urothelial carcinoma, creating a significant diagnostic dilemma. Cystoscopic evaluation, however, revealed a normal bladder mucosa without erythema, lesions, or findings suggestive of carcinoma in situ; no biopsy was performed. The absence of an identifiable urothelial malignancy or bacterial infection, together with the temporal association with pembrolizumab and the patient's concurrent multisystem irAEs, supported an immune-related etiology. This case highlights the potential for ICI-associated cystitis to produce cytologic atypia that closely mimics high-grade urothelial carcinoma and underscores the importance of integrating cytomorphologic findings with clinical history, radiologic and cystoscopic findings, microbiologic studies, and treatment history to avoid misdiagnosis. Recognition of this rare irAE is essential for appropriate management and prevention of unnecessary interventions.

## Introduction

Pembrolizumab is a programmed death receptor-1 (PD-1)-blocking monoclonal antibody that has become a cornerstone of treatment in advanced non-small-cell lung cancer (NSCLC) and is also used across multiple other malignancies, including bladder, renal cell, prostate cancer, melanoma, and other solid tumor settings [1,2]. By disrupting the PD-1/programmed death-ligand 1 (PD-L1) axis, pembrolizumab restores T-cell-mediated antitumor immunity, but this same mechanism may also impair peripheral immune tolerance and generate immune-related adverse events (irAEs) involving virtually any organ system [1,2]. In routine practice, the most familiar irAEs affect the skin, gastrointestinal tract, endocrine organs, liver, and lung, whereas genitourinary toxicities remain far less well characterized [1,2].

Among these uncommon toxicities, immune-related cystitis is particularly challenging because its true prevalence remains undefined. The available literature does not support a denominator-based incidence estimate for pembrolizumab-associated cystitis; rather, this entity has been described predominantly in isolated case reports, small case series, and narrative reviews [1-5]. Contemporary reviews explicitly note that bladder and ureter irAEs have generally only been described in case reports and are likely underrecognized, suggesting that the true burden is underestimated [1,2]. Published pembrolizumab-associated cases remain few, and the cumulative literature continues to evolve [3-5]. Accordingly, the most accurate statement at present is that pembrolizumab-associated cystitis is very rare and likely underrecognized, rather than a toxicity with a validated incidence estimate [1-5].

Clinically, pembrolizumab-related cystitis most often presents with hematuria, dysuria, urinary frequency, urgency, pollakiuria, painful micturition, pyuria, or pelvic discomfort, often in the setting of repeatedly negative urine cultures [3-5]. Radiographic findings may include bladder wall thickening, peri-bladder inflammatory change, or upper urinary tract involvement, whereas cystoscopy may demonstrate diffuse erythema, edema, inflammatory lesions, erosive change, or ulcerative remodeling [3-5]. Because these features overlap substantially with common urologic diagnoses, the differential diagnosis must be broad and systematic. At minimum, clinicians should exclude bacterial urinary tract infection, radiation-related cystitis, primary urothelial malignancy, malignant infiltration or metastasis involving the bladder, calculous disease, catheter-associated trauma, and other structural causes of hematuria before favoring an immune-mediated process [3-5]. In the absence of standardized diagnostic criteria, the most reproducible clinicopathologic framework includes checkpoint inhibitor exposure, new urinary symptoms and/or hematuria, sterile or repeatedly negative microbiologic studies, supportive imaging or cystoscopic evidence of inflammation, exclusion of malignant and alternative etiologies, and clinical improvement after corticosteroid therapy and/or treatment interruption [3-5].

Pathology has a central role in this diagnostic pathway, not because it is pathognomonic by itself, but because it helps exclude urothelial malignancy and documents an inflammatory injury pattern compatible with immune-mediated disease [3-5]. Routine histologic findings reported in pembrolizumab- and other immune checkpoint inhibitor (ICI)-associated cystitis include lymphocyte-predominant inflammatory infiltration, interstitial edema, epithelial desquamation or mucosal erosion, capillary proliferation, vasculo-exudative ulcerative remodeling, plasma cells, eosinophils, fibroblastic or granulation tissue change, and focal lymphoid follicle formation [3-5]. When immunohistochemistry has been performed, infiltrates enriched for CD8-positive and/or T-cell intracytoplasmic antigen-1 (TIA-1)-positive lymphocytes have been described, and some reports have also shown PD-L1 expression in the urothelium [3-5]. Importantly, routine hematoxylin-eosin findings are often nonspecific and may resemble severe chronic or interstitial cystitis; accordingly, biopsy results must be interpreted together with microbiology, imaging, cystoscopy, temporal association with pembrolizumab exposure, and treatment response [3-5].

This issue is directly relevant to the present case, in which pembrolizumab exposure in a patient with metastatic squamous NSCLC was followed by persistent hematuria and an acute cystitis picture on cross-sectional imaging, without urinary calculi or radiographic progression of pelvic metastases. Urine cytology showed atypical urothelial cells with high nuclear-to-cytoplasmic ratios and mild nuclear contour irregularities in a background of abundant acute inflammation and hemosiderin-laden macrophages, creating a diagnostically difficult distinction between reactive inflammatory atypia and clinically significant urothelial neoplasia. In this setting, cystoscopic and pathologic assessment is not merely confirmatory; it is central to excluding high-grade urothelial lesions and establishing whether the bladder findings are most consistent with an immune-mediated irAE. Given the expanding indications for pembrolizumab and the absence of standardized diagnostic criteria for immune-related cystitis, clinicians should maintain a high index of suspicion when patients receiving PD-1 blockade develop hematuria or persistent lower urinary tract symptoms without a clear infectious or structural explanation [1,2]. Early multidisciplinary evaluation involving oncology, urology, radiology, and pathology is essential to avoid delayed diagnosis, unnecessary antimicrobial exposure, and prolonged morbidity, while enabling timely corticosteroid-based management and informed decisions regarding immunotherapy interruption or continuation [1,2].

## Case presentation

A 55-year-old man with stage IV metastatic squamous cell carcinoma of the lung, with known sacral and brain metastases and a history of left upper lobectomy followed by chemotherapy, was receiving pembrolizumab-based systemic therapy. His clinical course was complicated by multiple irAEs involving several organ systems.

Approximately one year after initiation of pembrolizumab, he developed immune-mediated hypophysitis complicated by secondary adrenal insufficiency and thyroid dysfunction, evidenced by an 8:00 AM cortisol level of 1.6 µg/dL (~5-25 µg/dL) and a thyroid-stimulating hormone (TSH) of 8.8 mIU/L (~0.4-4.0 mIU/L). He was subsequently stabilized on hydrocortisone replacement and thyroid hormone therapy under the care of endocrinology. Several months later, he developed significant immune-mediated polyarthralgia requiring low-dose prednisone (5-10 mg daily) for symptom control.

Later in the course of pembrolizumab therapy, the patient developed intermittent gross hematuria, with laboratory evaluation demonstrating persistent microscopic hematuria. A CT scan of the abdomen and pelvis subsequently demonstrated an *acute cystitis picture* (Figure 1). Importantly, there was no evidence of urinary calculi or progression of pelvic metastatic disease that could otherwise account for his symptoms.

**Figure 1:**
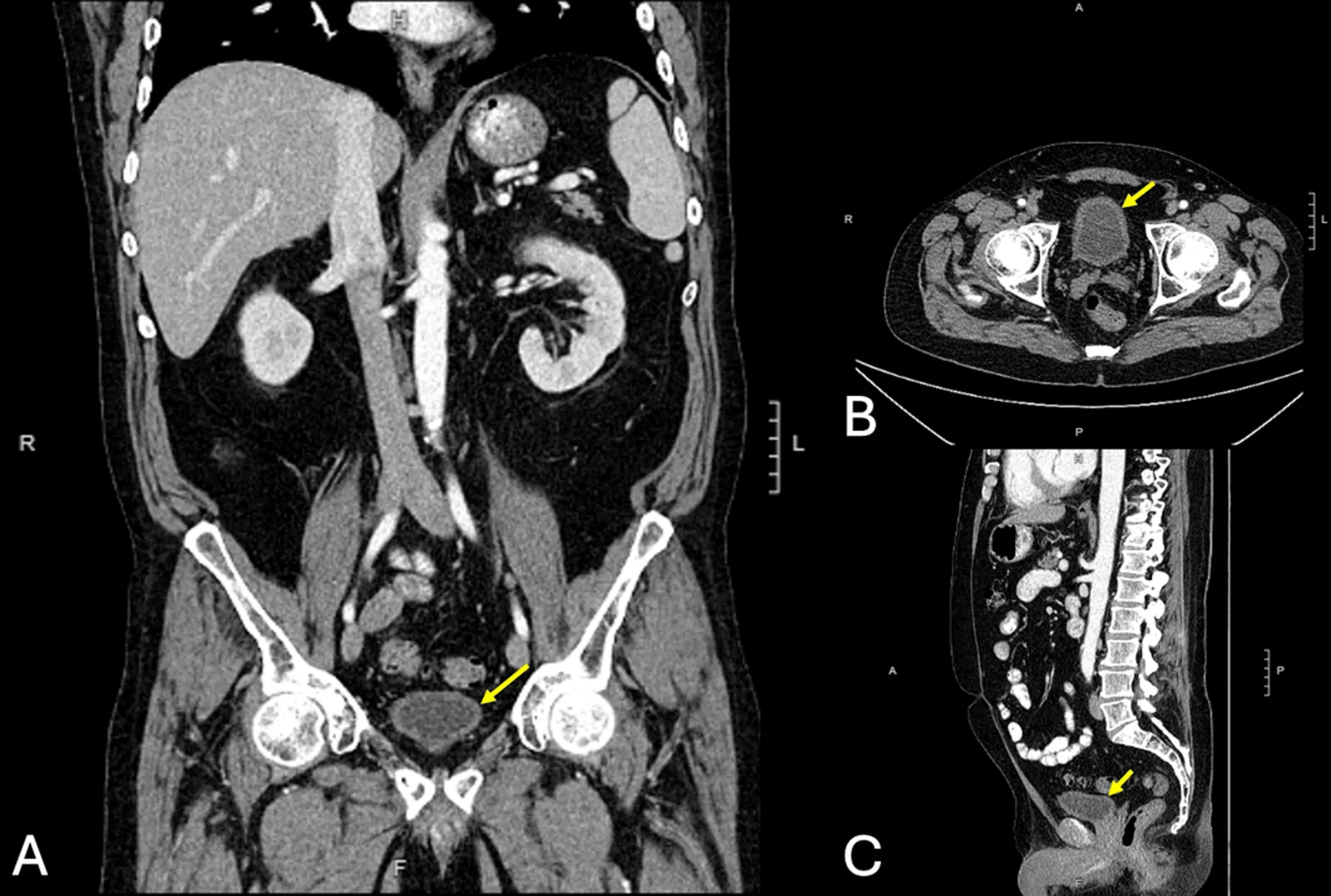
Contrast-enhanced computed tomography (CT) of the abdomen and pelvis in a 55-year-old man receiving pembrolizumab, presenting with hematuria. (A) Coronal reformatted image demonstrating minimal, relatively uniform bladder wall thickening (yellow arrow), without focal mass, ascites, or fluid collection. The kidneys enhance symmetrically with no hydronephrosis or upper-tract dilatation. (B) Axial image at the level of the bladder showing mild diffuse bladder wall thickening (yellow arrow) consistent with an inflammatory (*acute cystitis*) appearance; no discrete intraluminal mass, filling defect, or perivesical fat stranding suggestive of malignancy. (C) Sagittal reformatted image confirming mild bladder wall thickening (yellow arrow) and a mildly enlarged prostate, findings that may also reflect chronic bladder outlet obstruction. No pelvic lymphadenopathy or metastatic disease is identified.

Urine cytology performed using the ThinPrep® method (Hologic, Marlborough, MA) demonstrated atypical urothelial cells characterized by high nuclear-to-cytoplasmic ratios and mild nuclear contour irregularities, accompanied by abundant acute inflammation and hemosiderin-laden macrophages (Figure 2). The degree of urothelial atypia raised concern for a possible high-grade urothelial neoplasm, creating an important diagnostic dilemma in the setting of hematuria.

**Figure 2:**
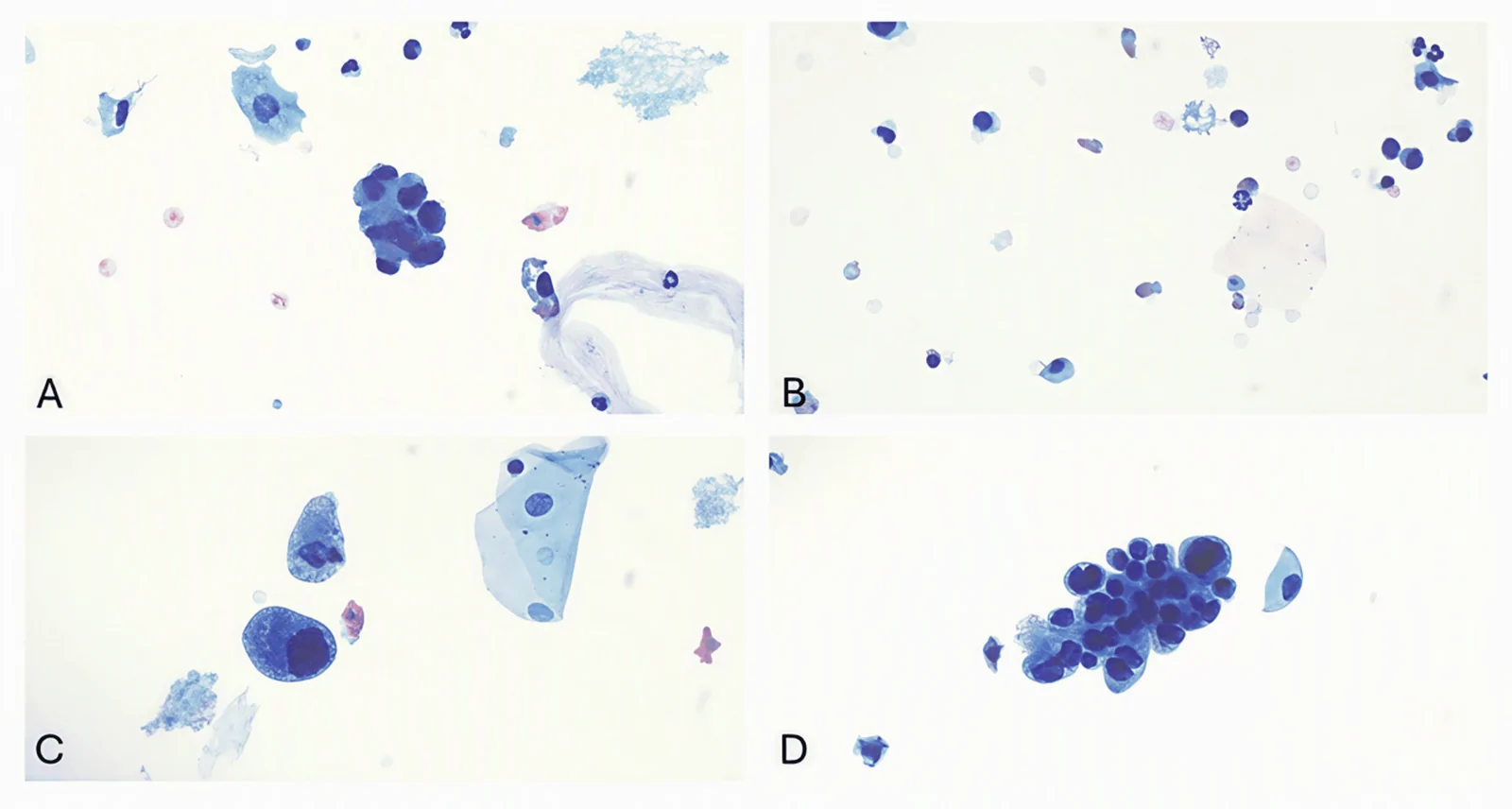
Voided urine cytology (ThinPrep preparation, Papanicolaou stain; original magnification ×600 for all panels) from a 55-year-old man receiving pembrolizumab, showing reactive urothelial atypia in a background of inflammation. (A) A cohesive, three-dimensional cluster of urothelial cells with enlarged, hyperchromatic nuclei and increased nuclear-to-cytoplasmic ratios, admixed with scattered neutrophils, degenerated cells, and background debris; a benign anucleate/squamous fragment and erythrocytes are also present. (B) Scattered atypical urothelial and inflammatory cells with occasional degenerative change in a clean-to-inflammatory background, including neutrophils and rare macrophages. (C) Individually dispersed atypical urothelial cells with nuclear enlargement and mild nuclear contour irregularity, together with a hemosiderin-laden macrophage and benign superficial squamous cells, consistent with prior hemorrhage in an inflammatory background. (D) A dense, overlapping cluster of urothelial cells with high nuclear-to-cytoplasmic ratios, nuclear crowding, and hyperchromasia, raising concern for high-grade urothelial carcinoma; however, the marked inflammatory background, temporal association with pembrolizumab, and subsequent normal cystoscopy favored reactive/immune-mediated atypia over malignancy.

Given the temporal association with pembrolizumab, the radiologic findings of acute cystitis, the absence of an alternative structural explanation, and the patient's established history of multisystem irAEs involving the pituitary-adrenal axis, thyroid, and joints, immune-related cystitis was strongly suspected.

The patient subsequently underwent flexible cystoscopy for further evaluation of the hematuria and to exclude an underlying urothelial malignancy. Cystoscopy demonstrated a normal urethra and bilobar prostatic enlargement without a median lobe. The bladder mucosa was entirely unremarkable, with no trabeculation, diverticula, focal lesions, patchy erythema, or other abnormalities suggestive of carcinoma in situ. The ureteral orifices were normal in number and configuration. No bladder biopsy or other specimen was obtained. Thus, cystoscopy did not demonstrate a urothelial lesion to account for the atypical cytologic findings.

Following cystoscopy, the urology team recommended clinical follow-up with repeat evaluation in six months. At the subsequent follow-up, the patient remained clinically well, with normal urinalysis/urine evaluation and an unremarkable urologic examination, without recurrent hematuria or other evidence of urinary tract malignancy. This subsequent clinical course further supported a reactive/inflammatory process rather than an underlying high-grade urothelial neoplasm.

At the time of follow-up, the patient remained on active systemic therapy with ramucirumab and pembrolizumab. His immune-mediated endocrine toxicities continued to be managed by endocrinology, including hydrocortisone replacement administered twice daily, with an additional oral morning dose as needed, and ongoing management of his thyroid dysfunction. Despite continued ICI therapy, there was no documented recurrence of hematuria or development of a structural bladder abnormality.

Overall, the combination of urothelial atypia mimicking high-grade urothelial carcinoma, radiologic evidence of acute cystitis, negative cystoscopy, subsequent resolution of urinary abnormalities, absence of an alternative structural explanation, and concurrent multisystem immune toxicities supported the diagnosis of pembrolizumab-associated immune-related cystitis with reactive urothelial atypia.

## Discussion

This case illustrates a clinically important *poly-irAE* phenotype, in which urological toxicity emerged in parallel with endocrine and musculoskeletal irAEs. This pattern matters because ICI toxicities may involve multiple organ systems, and the presence of established irAEs should raise suspicion that a new sterile inflammatory bladder syndrome may also be immune-mediated rather than infectious, structural, or malignant in origin [2]. In the present case, the coexistence of multi-organ toxicity strengthened the clinical plausibility of pembrolizumab-associated cystitis and helped frame the urinary findings as part of a broader immune dysregulation phenotype rather than an isolated urologic event [1,2].

A review of existing literature reveals a heterogeneous presentation of pembrolizumab-related cystitis, with latency periods ranging from early onset after several treatment cycles to approximately 11 months post-initiation or cumulative exposure (Table 1) [2,5]. Reported manifestations include hematuria, dysuria, urinary frequency, urgency, painful micturition, pyuria, pelvic discomfort, and, in more extensive cases, ureteral involvement with hydronephrosis or renal dysfunction [2,5]. The most consistent diagnostic pattern across published cases is the combination of persistent urinary symptoms, repeatedly negative or sterile microbiologic studies, supportive imaging or cystoscopic evidence of inflammation, and biopsy findings compatible with non-bacterial inflammatory cystitis [1,5]. Management has most commonly involved interruption or discontinuation of pembrolizumab and corticosteroid-based therapy, although diagnostic or supportive urologic procedures were required in selected cases when malignancy, bleeding, obstruction, or upper tract involvement had to be addressed [2,5].

**Table 1:** Summary of published cases of pembrolizumab-associated cystitis. TTF1, thyroid transcription factor 1; TURBT, transurethral resection of bladder tumor; EGFR, epidermal growth factor receptor; PET, positron emission tomography; NSCLC, non-small-cell lung cancer

Study/author (year)	Patient demographics	Clinical presentation	Time to onset	Diagnostic findings	Management	Outcome
El Husseini et al. (2021) [1]	56-year-old woman with metastatic TTF1-positive poorly differentiated adenocarcinoma/multimetastatic NSCLC treated with pembrolizumab 200 mg every three weeks	Hematuria, urinary burning, urgent polyuria, increasingly painful urination, and concurrent afebrile grade III diarrhea	After five cycles of pembrolizumab	Sterile urine with highly inflammatory cytology and high protein content; uroscanner showed thickened and irregular bladder walls; cystoscopy revealed highly inflammatory and irregular bladder mucosa; pathology showed diffuse inflammation with vasculo-exudative ulcerative remodeling; malignant cells were not identified	Pembrolizumab was suspended; systemic corticosteroids were administered at 1 mg/kg/day.	Clinical and radiologic response; because of thoracic progression, oncologic treatment was later resumed with paclitaxel-bevacizumab
Yan et al. (2026), Case 1 [2]	67-year-old woman with lung adenocarcinoma with brain metastasis and EGFR 19del mutation; previously treated with osimertinib-based therapy and later received pembrolizumab-containing therapy	Hematuria, dysuria, and urinary frequency; later recurrence with gross hematuria, worsened urinary frequency and urgency, fever, abdominal distension, and decreased urine output	Developed four days after treatment initiation following a cumulative total of three pembrolizumab cycles; recurrence occurred eight months after pembrolizumab discontinuation	Urinalysis showed elevated white and red blood cells; serum creatinine increased during the episode; follow-up CT showed persistent bilateral upper ureteral dilation with wall thickening, supporting cystitis with ureteritis after exclusion of alternative causes	Oral prednisone 40 mg daily, increased to 80 mg daily and tapered over 12 weeks; antitumor therapy was discontinued; during recurrence, intravenous methylprednisolone 40 mg daily was followed by oral prednisone 40 mg daily with weekly taper; pembrolizumab was permanently discontinued	Symptoms resolved rapidly with corticosteroids; serum creatinine normalized; persistent hematuria and residual ureteral dilation were noted after the initial episode; the patient later died from tumor progression two months after the recurrent episode
Yan et al. (2026), Case 3 [2]	35-year-old woman with endometrial clear cell carcinoma, stage IIIA; treated surgically, followed by albumin-bound paclitaxel/carboplatin; pembrolizumab was added after 2 cycles, and pelvic radiotherapy was given while immunotherapy continued	Urinary frequency, urgency, dysuria, and fever, followed by gross hematuria and flank pain	After four cycles of immunotherapy	Bilateral hydronephrosis and elevated serum creatinine were present; differential diagnosis included radiation cystitis and infectious etiologies; the clinical course supported immune-related cystitis and ureteritis.	Dexamethasone 5 mg daily and antibiotics for seven days; bilateral ureteral stent placement; intravenous methylprednisolone 80 mg daily followed by 40 mg daily; one session of methylprednisolone bladder irrigation was attempted but not tolerated; oral prednisone 40 mg daily was tapered weekly over eight weeks	By day 12 of corticosteroid therapy, urinalysis normalized; the patient became clinically stable and was discharged; pembrolizumab was resumed more than one month after discharge without recurrence of cystitis or ureteritis.
Di et al. (2024) [3]	A 27-year-old woman with driver gene-negative lung adenocarcinoma, cT4N3M1c stage IVB, treated with pembrolizumab-combined chemotherapy and later pembrolizumab-containing second-line therapy	Urinary frequency, hematuria, and painful micturition without fever	Urinary tract irritation appeared after the first course of second-line therapy; the discussion describes onset after approximately 11 months of pembrolizumab exposure	Multiple urine studies were negative for bacteria, fungi, and Mycobacterium tuberculosis; urine cytology was negative for high-grade urothelial carcinoma; imaging showed a bladder wall abnormality; cystoscopy showed diffuse congestion, edema, and thickening; TURBT biopsy showed chronic inflammatory cystitis with lymphocytes, plasma cells, eosinophils, edema, capillary proliferation, and focal lymphoid follicle formation; differential diagnosis included bacterial infection, fungal infection, tuberculosis, high-grade urothelial carcinoma, bladder metastasis, and IgG4-related sclerosing disease.	Cefoperazone/sulbactam was initially administered; TURBT was performed for diagnostic clarification; pembrolizumab was continued for a period but was later replaced by alectinib instead of pembrolizumab therapy and chemotherapy.	Bladder irritation improved shortly after TURBT, recurred after pembrolizumab was administered again, and disappeared after pembrolizumab was replaced.
He et al. (2022) [4]	56-year-old man with squamous carcinoma of the lung, staged as T1cN3M0 stage IIIB, treated with pembrolizumab after a plan for synchronous chemoradiotherapy	Frequent urination, urgent urination, micturition pain, pyuria, and hematuria	Symptoms developed on January 25, 2021, shortly after the sixth cycle of pembrolizumab given on January 16, 2021; pembrolizumab dose was not reported	Differential considerations included chronic cystitis, benign prostatic hyperplasia, and urinary infection; cystoscopic biopsy showed no malignant cells	Ceftriaxone for six days, followed by corticosteroid therapy with methylprednisolone 40 mg for three days and maintenance prednisolone	Urinary symptoms improved with corticosteroids; symptoms recurred after the seventh and eighth cycles of pembrolizumab and disappeared after one week of steroid therapy.
Alhusari et al. (2024) [5]	A 71-year-old man with stage II-B lung adenocarcinoma diagnosed six months earlier; history of chronic indwelling Foley catheter; received carboplatin/pemetrexed/pembrolizumab followed by maintenance pembrolizumab.	Worsening gross hematuria with dark red urine, weakness, shortness of breath, and anemia requiring blood transfusion	Gross hematuria began approximately one week before admission; the last dose of the fourth cycle of pembrolizumab was given one week before admission; pembrolizumab dose was not reported.	Persistent hematuria despite antibiotics for presumed urinary infection; cystoscopy did not show bladder masses or an active bleeding artery; bacterial cystitis and structural malignant lesions were not supported	Antibiotics, cystoscopy, suprapubic catheter placement, prednisone 1 mg/kg, temporary discontinuation of pembrolizumab, and trimethoprim-sulfamethoxazole for Pneumocystis jirovecii pneumonia prophylaxis	Hematuria improved at hospital discharge follow-up; steroid taper was initiated; PET imaging later showed disease progression, and systemic therapy with pemetrexed and carboplatin was started.

For clinicians, pembrolizumab-associated cystitis should be approached as a diagnosis of exclusion rather than a diagnosis made from symptoms alone. The first step is to establish a clear temporal relationship between pembrolizumab exposure and the onset of urinary symptoms, including treatment cycle, cumulative duration, prior irAEs, catheter use, prior pelvic radiation, recent antimicrobial therapy, and tumor status [2,5]. Infection should then be excluded with urinalysis and urine cultures, with repeat microbiologic testing when symptoms persist despite empiric antibiotics or when pyuria and hematuria remain unexplained [5]. Imaging is useful to assess for bladder wall thickening, perivesical inflammatory change, hydronephrosis, ureteral dilatation, stones, clot burden, obstruction, or tumor-related involvement [2,5]. Persistent hematuria, abnormal cytology, severe symptoms, or suspicious imaging should prompt early urologic assessment because cystoscopy and biopsy may be required to exclude primary urothelial carcinoma, malignant infiltration, radiation cystitis, calculous disease, catheter-associated trauma, or another structural explanation [1,5].

Pathology and cytology must be interpreted cautiously in this setting because immune-mediated cystitis can produce marked inflammatory and reactive changes without being pathognomonic. Reported histologic findings include lymphocyte-predominant inflammation, interstitial edema, epithelial desquamation, mucosal erosion, plasma cells, eosinophils, capillary proliferation, granulation tissue change, and focal lymphoid follicle formation [1,5]. When immunohistochemistry has been performed, CD8-positive and/or TIA-1-positive lymphocytic infiltrates have been described, supporting a cytotoxic T-cell-mediated pattern of injury in selected cases [1,5]. In the present case, urine cytology showed atypical urothelial cells with high nuclear-to-cytoplasmic ratios and mild nuclear contour irregularities in a markedly inflammatory background with hemosiderin-laden macrophages. Diagnostic challenges are further compounded by the interpretation of urine cytology in the setting of immune-mediated inflammation. According to The Paris System for Reporting Urinary Cytology, reactive changes such as those seen in immunotherapy-induced cystitis can easily mimic high-grade urothelial carcinoma (HGUC). The presence of enlarged nuclei, hyperchromasia, and prominent nucleoli within reactive urothelial cells may be erroneously classified as *Atypical Urothelial Cells *or even *Suspicious for HGUC*. Therefore, cytopathologists must exercise caution; a diagnosis of *Atypical* in this context should explicitly comment on the inflammatory background and the temporal correlation with immunotherapy. Accordingly, atypical urine cytology in a patient receiving pembrolizumab should be integrated with the inflammatory background, cystoscopic appearance, histopathology, imaging, microbiologic studies, and clinical course rather than interpreted as independent evidence of malignancy [5].

Management should be individualized according to symptom severity, renal function, extent of urinary tract involvement, oncologic status, and diagnostic certainty. In confirmed or strongly suspected cases, the current literature supports pembrolizumab interruption and corticosteroid-based therapy as the core interventions, particularly when infection and malignancy have been reasonably excluded [2,5]. Supportive and adjunctive measures may be necessary while the diagnostic workup is ongoing, including empiric antibiotics when infection remains plausible, catheter management or bladder irrigation for significant hematuria, transurethral biopsy or resection when malignancy cannot be excluded, and ureteral stenting or nephrostomy when obstruction threatens renal function [2,5]. Because recurrence has been described after continued exposure, rechallenge, and delayed post-discontinuation periods, follow-up should include reassessment of urinary symptoms, urinalysis, renal function when upper tract involvement is suspected, and repeat imaging or cystoscopy when recovery is incomplete, or the clinical course is atypical [2,5]. A multidisciplinary pathway involving oncology, urology, radiology, pathology, and nephrology when appropriate is essential to reduce unnecessary antimicrobial exposure, avoid delayed diagnosis, preserve urinary tract function, and guide safe decisions regarding pembrolizumab interruption, discontinuation, or carefully selected resumption [2,5].

## Conclusions

Immune-related cystitis is a rare but clinically important genitourinary toxicity of ICI therapy that should be considered in patients presenting with hematuria, dysuria, or other urinary symptoms, particularly in the setting of sterile cultures, compatible imaging findings, and concurrent multisystem irAEs. This case highlights an important cytopathologic pitfall: reactive urothelial atypia associated with pembrolizumab-associated cystitis may closely mimic high-grade urothelial carcinoma on urine cytology. Careful integration of cytologic, radiologic, cystoscopic, microbiologic, and clinical findings is therefore essential to distinguish immune-mediated inflammation from malignancy and infection. Early recognition and multidisciplinary evaluation can facilitate appropriate management, minimize unnecessary interventions, and support individualized decisions regarding continuation or interruption of immunotherapy.
